# Electrical storm 11 days after acute myocardial infarction: a case report

**DOI:** 10.1186/s13256-019-2267-5

**Published:** 2019-11-27

**Authors:** Sayaka Ohsawa, Hiroki Isono, Eiji Ojima, Masahiro Toyama, Yasuhisa Kuroda, Shigeyuki Watanabe, Toshikazu Abe

**Affiliations:** 1 Department of Internal Medicine, Kitaibaraki City Hospital, 1050 Sekimotoshita, Sekinan, Kitaibaraki, Ibaraki, Japan; 2 Department of General Medicine and Primary Care, Tsukuba University Hospital, 2-1-1 Amakubo, Tsukuba, Ibaraki, 305-8576 Japan; 3 Department of Cardiology, Mito Kyodo General Hospital, 3-2-7 Miyamachi, Mito, Ibaraki, Japan; 4 Department of General Medicine, Juntendo University, 2-1-1 Hongou, Bunkyo, Tokyo, Japan

**Keywords:** Cardiopulmonary arrest, Circulation, Acute myocardial infarction, Electrical ablation, Overdrive pacing

## Abstract

**Background:**

The definition of electrical storm is still debated. For example, an electrical storm is defined as a clustering of three or more separate episodes of ventricular tachycardia/ventricular fibrillation within 24 hours or one or more episodes occurring within 5 minutes of termination of the previous episode of ventricular tachycardia/ventricular fibrillation. When it is refractory to medications, prompt assessments by coronary angiography, sedation, and overdrive pacing should be performed. An electrical storm may occur anytime, including at night or after the patient leaves an intensive care unit.

**Case presentation:**

A 70-year-old Japanese man with type 2 diabetes mellitus was diagnosed as having ST-elevation myocardial infarction. His clinical course after an urgent percutaneous coronary intervention was uneventful, but he developed electrical storm that was refractory to antiarrhythmic medications on day 11 of hospitalization. We used sedative medications and performed ventricular overdrive pacing and transferred him to a university hospital for further treatment, which included electrical ablation and cardioverter-defibrillator implantation.

**Conclusion:**

An electrical storm is a relatively rare and fatal complication of acute myocardial infarction. It is important that the treatment choices for this condition are known by non-cardiologist physicians who might encounter this rare condition.

## Background

The definition of electrical storm (ES) varies, for instance, it is defined as a clustering of three or more separate episodes of ventricular tachycardia (VT)/ventricular fibrillation (VF) within 24 hours [1] or one or more episodes occurring within 5 minutes of termination of the previous episode of VT/VF [2]. There are various causes of ES, such as structural heart diseases, electrolyte imbalances, drug effects, and prolonged QT; an ES can make a patient hemodynamically unstable and present as a life-threatening complication of acute myocardial infarction (AMI). Patients repeatedly go into VT/VF, receive antiarrhythmic medications serially, and undergo repeated electrical shocks in an attempt to cardiovert the arrhythmia. Some ESs are refractory to drug treatment and repetitive cardioversion is harmful to heart muscle cells [3–5]. Therefore, other prompt assessments such as a second coronary angiography (CAG) and other treatments, such as sedation and overdrive pacing, should be administered. We present our experience in a case of ST-elevation myocardial infarction (STEMI) with ES that occurred on the night of day 11 of hospitalization when the cardiologist was not in the hospital and the case was refractory to intravenously administered therapy and needed other forms of treatment.

## Case presentation

**Fig. 1:**
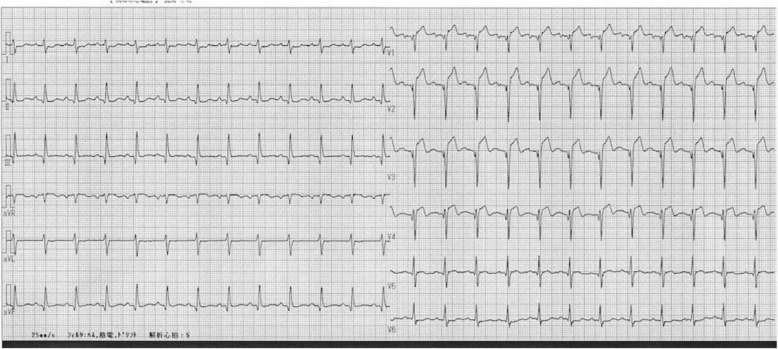
Electrocardiogram on admission

**Fig. 2:**
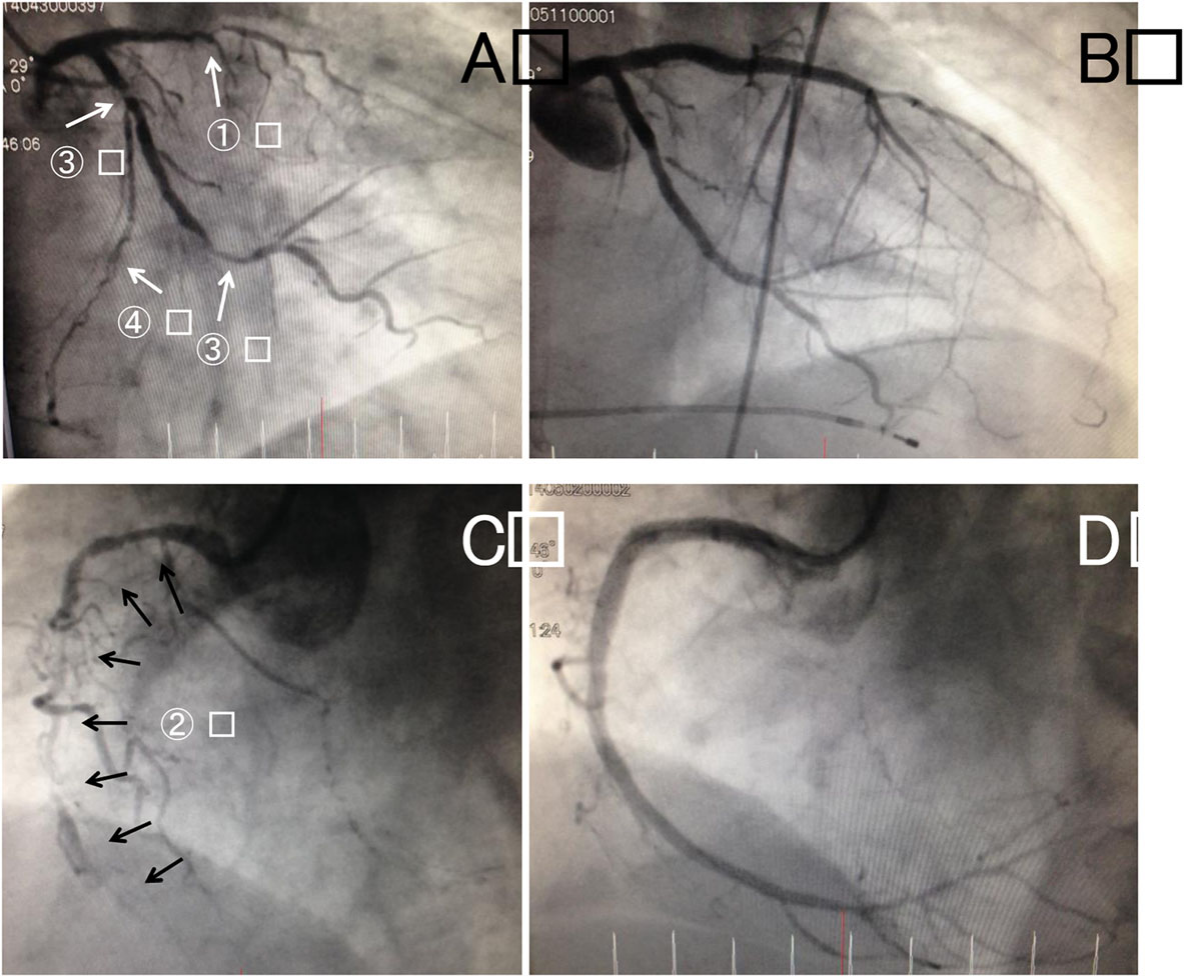
Coronary angiograms. The coronary angiograms of the left coronary (**a**) and right coronary arteries (**c**). A totally occluded lesion in the proximal left anterior descending artery ① was treated with drug-eluting stent on the day of hospitalization. The diffuse stenosis in the right coronary artery ② was also treated with drug-eluting stents on the following day. The remaining lesions of the proximal and middle segments of the left circumflex artery were treated with drug-eluting stents on day 7 of hospitalization ③. Coronary angiograms after the successive treatments (**b** and **d**). Note the disappearance of the collateral vessel ④ to the right coronary artery via the atrial circumflex branch of the left circumflex artery after successful revascularization of the right coronary artery

A 70-year-old Japanese man with complaints of abdominal bloating for 2 weeks along with liver dysfunction and elevated C-reactive protein (CRP) levels was referred to our hospital. He denied having chest pains but reported that his abdominal bloating worsened 6 hours before his arrival at our hospital. His medical history included type 2 diabetes mellitus and benign prostatic hyperplasia. He did not have any medical history of cardiovascular disease or arrhythmias. Both his parents were diagnosed as having diabetes mellitus. He lived with his wife and son and retired from a metal processing company approximately 10 years ago. His routine medications were silodosin 8 mg, distigmine 4 mg, pioglitazone 30 mg, dutasteride 0.5 mg, simvastatin 5 mg, lansoprazole 30 mg, glimepiride 1 mg, and rebamipide 100 mg. He did not consume alcohol but had a 100 pack-year smoking history. His blood pressure was 112/67 mmHg, pulse rate was 111 beats per minute (bpm), respiratory rate was 16 breaths/minute, body temperature was 37.0 °C, and oxygen saturation was 98% while breathing ambient air. There were no abnormal findings on cardiorespiratory, abdominal, and neurological examinations. Laboratory findings included the following: blood urea nitrogen (BUN)/creatinine (Cre) 27/1.22 mg/dL, aspartate aminotransferase (AST)/alanine aminotransferase (ALT) 72/54 IU/L, alkaline phosphatase (ALP)/gamma-glutamyltransferase (GGT) 366/128 IU/L, total bilirubin (T-bil) 2.4 mg/dL, CRP 21.9 mg/dL, creatine kinase (CK) 631 IU/L, and positive troponin T (only the qualitative test was available). A screening electrocardiogram (ECG) on admission showed ST segment elevation in leads V1–4 and an abnormal Q wave in leads II, III, aVF, and V1–4 (Fig. 1). Transthoracic echocardiography revealed anterior wall akinesis and ejection fraction (EF) of 30%. Abdominal echocardiography showed no abnormality. He was diagnosed as having STEMI without any chest pain. We considered the condition to be in the very acute phase (< 12 hours) of myocardial infarction (MI), because the ST segment elevation was very intense and was accompanied by tall T waves, and no T wave inversions were observed in any leads. Therefore, we performed urgent CAG and it revealed triple-vessel disease with total occlusion in the proximal left anterior descending artery along with 90–99% diffuse stenosis in the proximal to middle segments of the right coronary artery, and 90% stenosis in both proximal and middle segments of the left circumflex artery (Fig. 2).

**Fig. 3:**
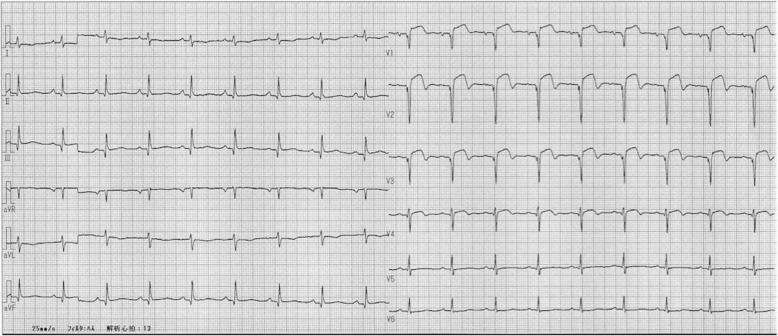
Electrocardiogram after percutaneous coronary interventions (day 8)

A drug-eluting stent (DES) was placed in the proximal left anterior descending artery, which was the site of the culprit lesion. Our patient was supported with an intra-aortic balloon pump. On day 2 of hospitalization, the proximal and middle segments of the right coronary artery were also successfully treated with DES, and, on day 7, both the proximal and middle segments of the left circumflex artery were also treated. An ECG after percutaneous coronary intervention is shown in Fig. 3.

**Fig. 4:**
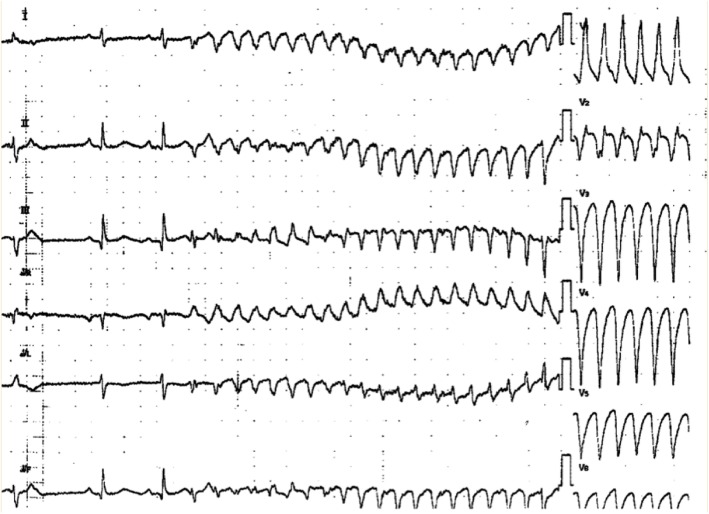
Electrocardiogram at the onset of electrical storm

We prescribed two antiplatelet drugs, which were Bayaspirin (aspirin) 100 mg and clopidogrel 75 mg, a statin (atorvastatin 10 mg), an angiotensin converting enzyme inhibitor (enalapril 2.5 mg), and a beta-blocker (carvedilol 1.25 mg which was increased to 2.5 mg on day 3 after we checked the tolerance) to our patient. His peak CK level was 1741 U/L. His clinical course was uneventful, so cardiac rehabilitation was started on day 5 of hospitalization. However, he suddenly developed pulseless VT at night on day 11 (Fig. 4). He was still in the intensive care unit (ICU) because the general ward was at full capacity. He was successfully resuscitated with defibrillation during the first VT. Blood tests including electrolyte levels were normal, except for potassium level that was 4.05 mEq/L, which was lower than the recommended target range for potassium after AMI [6]. Therefore, potassium was slowly infused intravenously to achieve the target range. No QT prolongation or ST segment elevation was observed on ECG. No signs of acute heart failure were noted. However, a second episode of pulseless VT recurred after 50 minutes, and it was followed by repeated episodes of VT and VF. He was diagnosed as having an ES. Initial rapid administration of 125 mg amiodarone was started after the second episode and an additional dose of amiodarone as loading after later subsequent events and 2 g of magnesium were started to resolve ES, but it persisted. Cardiology consultation was requested, but it was not possible for the cardiologist to arrive within an hour. Continuous infusion of landiolol and 125 mg of Mexitil (mexiletine hydrochloride) in succession was carried out, but they were ineffective. Midazolam for sedation was also administered for sedation. Ultimately, more than 50 defibrillations were needed. The second CAG showed no stenosis. A temporal intravenous pacemaker was inserted and set up for overdrive pacing at 120 bpm to suppress ES. No more episodes of ES were detected after overdrive pacing was initiated. After hemodynamic stabilization was achieved, our patient was transferred to a university hospital to be considered for electrical ablation and a cardioverter-defibrillator implantation. Electrical ablation was performed successfully, and he has had no further episodes of ES to date. He visits our hospital regularly; implantable cardioverter defibrillator (ICD) was reimplanted 5 years later.

## Discussion and conclusions

Here we report a case of drug-refractory ES that occurred on the night of day 11of hospitalization following STEMI, during the subacute phase of AMI treatment when only a brief over-the-telephone consultation with a cardiologist was possible. ES continued despite the use of antiarrhythmic drugs, sedatives, and beta-blockers to control sympathetic nerve activity. The possible mechanisms for ES after AMI include triggered activity, abnormal automaticity, microre-entry, and transmural re-entry [4]. Activated automaticity is worsened by sympathetic nerve activity [7]; therefore, beta-blockers, stellate ganglionic blockade, and sedative agents are recommended in addition to antiarrhythmic medications [8]. Although propranolol is an option for treatment of ES, we selected landiolol, a highly selective beta-blocker, because it has minimal effect on cardiac function [7, 9]. Direct cell injury caused by frequent delivery of defibrillator shocks [3–5] might result in high mortality rates. Therefore, other treatments should be administered promptly when attempted treatments are judged to be ineffective in resolving ES. In this case, overdrive pacing and subsequent ablation were successful. Our patient had no more occurrences of ventricular arrhythmia.

**Fig. 5:**
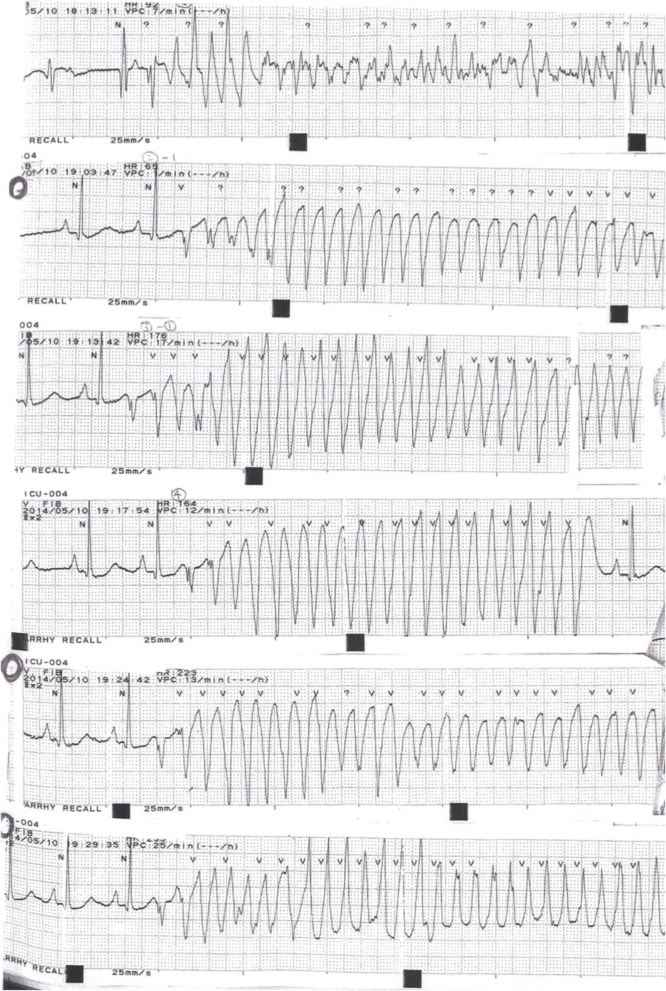
Electrocardiogram at another moment of electrical storm onset

Viskin *et al.* reported quinidine-responsive polymorphic VT in patients with coronary heart disease [10]. In their study, the polymorphic VT in patients with coronary heart disease shared important characteristics with idiopathic VF and Brugada syndrome; the characteristics are distinctive arrhythmia mode of onset by short-coupled extrasystoles and susceptibility to develop arrhythmic storms that are refractory to conventional antiarrhythmic drugs, including amiodarone. Our patient exhibited similar characteristics in an ECG (Fig. 5). They also found that this polymorphic VT in ischemic heart disease also responds to quinidine-like idiopathic VF and Brugada syndrome. Therefore, our patient might have responded to the quinidine treatment.

Transcutaneous cardiac pacing could be a treatment of choice among non-cardiologists, although there is a moderate risk of failure in achieving cardiac capture, which is one of its limitations [11]. Therefore, transcutaneous cardiac pacing should be limited as a bridging treatment for intravenous cardiac pacing only when access to a cardiologist is limited or untimely.

ES is a relatively rare complication of AMI that can lead to death when effective treatments are not immediately administered. However, the average duration of ES occurrence is approximately 11–12 days after AMI [8], which could be at daytime or nighttime, and after leaving an ICU. It has been reported that 58.7% of incidences of ES occur between 8 a.m. and 4 p.m., and the rest occur at night [12]. It is important that non-cardiologist physicians in charge of the night shift who might encounter this rare condition know the treatment choices, make treatment decisions, and act for consulting cardiologists because ESs do not only occur during the hyperacute phase of AMI but also during times of inadequate medical resources or when prompt contact with a cardiologist is not possible.

## Data Availability

Data sharing is not applicable to this article.

## Abbreviations

AMI: Acute myocardial infarction
bpm: Beats per minute
CAG: Coronary angiography
CK: Creatine kinase
CRP: C-reactive protein
DES: Drug-eluting stent
ECG: Electrocardiogram
EF: Ejection fraction
ES: Electrical storm
ICU: Intensive care unit
STEMI: ST-elevation myocardial infarction
VF: Ventricular fibrillation
VT: Ventricular tachycardia
